# The metabolic syndrome and progression of carotid atherosclerosis over 13 years. The Tromsø study

**DOI:** 10.1186/1475-2840-11-77

**Published:** 2012-06-27

**Authors:** Marit Herder, Kjell Arne Arntzen, Stein Harald Johnsen, Ellisiv B Mathiesen

**Affiliations:** 1 Department of Community Medicine, University of Tromsø, N-9038, Tromsø, Norway; 2 Department of Radiology, University Hospital North Norway, Tromsø, Norway; 3 Department of Neurology and Neurophysiology, University Hospital North Norway, Tromsø, Norway; 4 Department of Clinical Medicine, University of Tromsø, Tromsø, Norway

**Keywords:** Metabolic syndrome, Carotid artery, Atherosclerosis, Intima-media thickness, Plaque, Progression, Risk factor, Prospective, Population study

## Abstract

**Background:**

The metabolic syndrome (MetS) is associated with increased risk of cardiovascular disease. In this study, we examine if metabolic syndrome predicts progression of atherosclerosis over 13 years.

**Methods:**

Participants were 1442 men and 1532 women in the population-based Tromsø Study who underwent carotid ultrasound examinations at baseline in the 4^th^ (1994–5) and at follow-up in the 6^th^ survey (2007–8). Of these, 278 men and 273 women fulfilled the criteria for the MetS, defined according to a modified version of the National Cholesterol Education Program Adult Treatment Panel III (NCEP, ATPIII). Carotid atherosclerosis was assessed as total plaque area (TPA) and mean intima-media thickness (IMT) at follow-up and as change in IMT and TPA from baseline to follow-up. Associations between MetS and its components and carotid atherosclerosis were assessed in linear regression models adjusted for age, total cholesterol and daily smoking, stratified by sex.

**Results:**

IMT and TPA levels at follow-up (p < 0.0001) and progression of TPA (p = 0.02) were higher in the MetS group compared to the non-MetS group. In stepwise multivariable models, MetS was associated with TPA (β = 0.372 mm^2^, p = 0.009) and IMT (β = 0.051 mm, p < 0.0001) in men, and with IMT (β = 0.045 mm, p = 0.001) in women after 13 years of follow-up, but not with progression of IMT or TPA. In analyses stratified by age, MetS predicted progression of IMT (β = 0.043 mm, p = 0.046) and TPA (β = 1.02 mm^2,^ p = 0.002) in men below 50 years of age. Hypertension was predictive of follow-up TPA and IMT in both genders and of progression of TPA in women. Impaired glucose tolerance was associated with follow up levels of IMT and TPA as well as progression in IMT in men. None of the other components of MetS were associated with progression of atherosclerosis.

**Conclusions:**

Subjects with MetS had higher levels of IMT and TPA at follow up than those without MetS. Mets predicted progression of IMT and TPA in those below 50 years of age, but not in other age groups, indicating that MetS may be involved in the initiation of the atherosclerotic process.

## 

Metabolic syndrome (MetS) is a cluster of metabolic and non-metabolic cardiovascular risk factors, including insulin resistance, dyslipidaemia, visceral adiposity and hypertension. However, the pathophysiological basis and utility of MetS are debated, although several studies have shown associations between MetS and increased risk of cardiovascular disease (CVD) [1-7].

Atherosclerosis is the underlying process of a majority of cardiovascular disease and mortality. While the clinical manifestations of atherosclerosis usually do not occur until middle age, atherosclerosis develops early in life. Noninvasive ultrasonographic assessment of carotid intima-media thickness (IMT) and total plaque area (TPA) is suitable for evaluation of the burden of atherosclerosis, and are predictive of future risk of CVD. Although inter-correlated, measurements of IMT and TPA are thought to reflect different biological aspects of and stages in the development of atherosclerosis. Whereas TPA measures formed plaques, IMT can be measured where no focal disease is present. Both cross-sectional and prospective studies have shown association between MetS and IMT [1,3,8-10]. Data on associations between plaque measurements and MetS are scarce [6,9,11]. In a study on 166 members of the Canadian Oji-Cree community, a population with one of the world`s highest prevalence rates of the MetS, MetS was associated with IMT and total plaque volume after 7 years of follow-up [9]. In the prospective Bruneck study, subjects with MetS had higher progression of atherosclerosis as assessed by formation of new plaques and carotid stenosis [11]. In a cross-sectional study, plaque presence was associated with metS in women only [6].

In the prospective population-based Tromsø Study, we explored the relationship between MetS and progression of atherosclerosis in 2795 persons after 13 years. Information on MetS and cardiovascular risk factors were obtained at baseline. Carotid atherosclerosis, assessed as IMT, TPA and plaque number, was measured at baseline and at follow-up.

## Subjects and methods

### Subjects

The Tromsø Study is a longitudinal population-based health study with repeated surveys of the adult population in the municipality of Tromsø, Norway [12]. The study has been approved by the Regional Committee for Medical and Health Research Ethics, the Norwegian Directorate of Health and the Data Inspectorate.

Subjects eligible for the present study were those who participated in ultrasound examination in the 4^th^ (1994–1995) and 6^th^ survey (2007–2008) of the Tromsø Study. The 4^th^ survey consisted of two screening visits, and ultrasound examination of the carotid arteries was done at the 2^nd^ visit. All inhabitants of Tromsø aged 55–74 years and random 5-10% samples of subjects in the age groups 20–54 years and 75–84 years were invited to the 2^nd^ visit, and 6885 subjects attended (79% of the eligible population). Carotid ultrasound examination was performed in 6727 subjects. During follow-up, 1451 persons died and 486 moved from Tromsø. Forty-one subjects were excluded because they had withdrawn their written consent to further research. Of the remaining 4750 subjects who were still alive and living in Tromsø, 2974 subjects (62.6%) attended the carotid ultrasound examination in the 6^th^ survey in 2007–2008, and were included in the present study. All included participants gave informed, written consent.

### Baseline risk factors

At baseline, information on diabetes mellitus, use of insulin and/or anti diabetic drugs, smoking habits, history of cardiovascular diseases and treated hypertension (never/previous/current) were obtained from self-administered questionnaires. Height and weight were measured with subjects wearing light clothing and without shoes. BMI was calculated as weight in kilograms divided by squared height in meters (kg/m^2^). Waist circumference was measured at the umbilical line. Blood pressure was recorded three times at one-minute intervals after two minutes of seated resting with the use of an automatic device (Dinamap Vital Signs Monitor 1846, Criticon) and by specially trained technicians. The mean of the last two recordings was used in the report. Analyses of non-fasting serum total cholesterol and triglycerides were done using commercial kits. Serum high density lipoprotein (HDL) cholesterol was measured after the precipitation of lower-density lipoprotein with heparin and manganese chloride. The low density lipoprotein (LDL) concentration was calculated according to Friedewald’s formula: LDL-cholesterol = Total cholesterol – HDL-cholesterol – (0.45 x triglycerides) in 2961 subjects with triglyceride levels below 4.52 mmol/L. Lipid levels were measured twice with an interval of 4–12 weeks and the averages of these values were used in the analyses. Serum uric acid was measured by photometry with COBAS® instruments (Roche diagnostics, Switzerland) using an enzymatic colorimetric test, the uricase/PAP method. Glycosylated haemoglobin (HbA1C) levels were measured with a liquid chromatographic procedure. All analyses were performed at the Department of Clinical Chemistry, University Hospital of Northern Norway.

### Definition of metabolic syndrome

MetS was defined according to the National Cholesterol Education Program Adult Treatment Panel III (NCEP, ATPIII) [13]. According to this definition, the MetS is present when three or more of the following five criteria are fulfilled; abdominal obesity, hypertriglyceridemia, low HDL-cholesterol, hypertension, or elevated fasting glucose. Abdominal obesity is defined as waist circumference ≥ 102 cm in men and ≥ 88 cm in women. Hypertriglyceridemia is defined as elevated triglycerides ≥ 150 mg/dL (1.7 mmol/L) or self-reported lipid lowering drug treatment. Low HDL cholesterol is defined as < 40 mg/dL (1.0 mmol/L) for men and < 50 mg/dL (1.30 mmol/L) for women or self-reported lipid lowering drug treatment. As fasting glucose was not measured in the Tromsø Study, HbA1c ≥6.1% and/or non-fasting plasma glucose >11.1 mmol/L and/or self-reported diabetes and/or use of anti-diabetic medication was defined as impaired glucose tolerance. Hypertension was defined as elevated systolic blood pressure ≥ 130 mmHg, or diastolic blood pressure ≥ 85 mmHg, or self-reported current antihypertensive drug treatment [14].

### Carotid ultrasound measurements

High-resolution B-mode ultrasonography at baseline was performed with Acuson Xp10 128, ART-upgraded duplex scanners equipped with 7.5 MHz linear array transducers, while GE Vivid 7 duplex scanners with linear 12 MHz transducers were used at follow-up [15]. Subjects were examined in the supine position with the head slightly tilted to the opposite side. No fixed angle of insonation was used; the sonographers were instructed to view the arteries from all possible angles, in order to find the optimal view for visualization of plaque and IMT in each subject. The far- and near walls of the right common carotid artery (CCA), bifurcation (bulb) and internal carotid artery (ICA) (six locations) were scanned for the presence of plaques. A plaque was defined as a localized protrusion into the vessel lumen with thickening of the vessel wall of more than 50% compared to the adjacent IMT. The outline of each plaque was marked manually on still images, with calculation of plaque area. In subjects with more than one plaque, TPA was calculated as the sum of all plaque areas. Semi-automated ECG-triggered measurement of IMT was performed in 10 mm segments of the far (CCA-FW-IMT) and near wall (CCA-NW-IMT) of the CCA and in the most proximal 10 mm far wall segment of the bulb (BULB-FW-IMT). Mean IMT from the 3 pre-selected images was calculated for each location. If present in the predefined location of interest, plaques were included in the IMT measurements. The average of mean IMT from the three locations was used in the analyses (hereafter referred to as IMT). Final reading of IMT and plaque area was done off line using the automated Artery Measurement System II [16]. The inter- and intra-observer and inter-equipment reproducibility of IMT and plaque measurements was acceptable [15,17-19].

### Statistical analysis

Stata SE 11 (StataCorp LP, College Station, TX, USA) and the SAS software, version 9, were used for all analyses. Differences between subjects with and without MetS were analyzed using *t*-test (continuous variables) Wilcoxon rank-sum test and χ ² (dichotomous variables). Values are presented as means (SD) or numbers (%). TPA was square-root-transformed to approximate normal distribution. Changes in IMT and square-root-transformed TPA were calculated by subtracting the value at baseline from the follow-up value (ΔIMT and ΔTPA). Linear regression models were fitted with IMT and TPA as dependent variables and MetS, age, total cholesterol and smoking as independent variables. Similarly, stepwise linear multivariable models with forward selection and significance level 0.05 for entry into the model were fitted with each component of the metabolic syndrome entered as separate independent variables, together with age, total cholesterol and smoking. Interaction with sex was examined with IMT and TPA as the dependent variable and sex, risk factor, and sex*risk factor as independent variables. There was significant interaction between sex and MetS in the IMT models, all analyses were therefore stratified by sex. Further adjustments were made for uric acid and use of lipid-lowering, anti-platelet and antihypertensive drugs at baseline and follow-up. Two-sided p-values < 0.05 were considered statistically significant.

## Results

Baseline characteristics of the 273 women and 278 men who met the criteria for MetS are shown in Table 1. Women with MetS were older and fewer smokers than women without MetS. Subjects with MetS had increased IMT, more plaques and larger TPA at baseline (Table 1).

**Table 1:** Baseline characteristics* in subjects with and without metabolic syndrome, by sex

	**Women**			**Men**		
	**Metabolic syndrome**			**Metabolic syndrome**		
	**Yes**	**No**	**p**	**Yes**	**No**	**p**
Age	60.4 (7.3)	56.0 (10.4)	<0.0001	55.5 (8.3)	56.2 (9.2)	0.3
Systolic blood pressure (mmHg)	153.1 (20.9)	135.6 (20.7)	<0.0001	146.0 (16.2)	139.0 (18.1)	<0.0001
Diastolic blood pressure (mmHg)	86.3 (12.9)	77.9 (11.6)	<0.0001	87.3 (10.6)	82.6 (11.4)	<0.0001
Hypertension treatment (%)	59 (21.6)	80 (6.5)	<0.0001	44 (15.9)	73 (6.4)	<0.0001
Components of metabolic syndrome						
Waist circumference (cm)	93.8 (9.1)	81.35 (8.4)	<0.0001	102.5 (8.4)	92.7 (7.1)	<0.0001
Triglycerides (mmol/L)†	2.29 (0.87)	1.18 (0.54)	<0.0001	2.63 (1.04)	1.50 (0.74)	<0.0001
HDL (mmol/L)	1.37 (0.32)	1.75 (0.38)	<0.0001	1.10 (0.26)	1.43 (0.34)	<0.0001
Diabetes (%)	18 (6.6)	8 (0.7)	<0.0001	11 (4.0)	6 (0.5)	<0.0001
HbA1c %	5.68 (0.64)	5.35 (0.34)	<0.0001	5.47 (0.62)	5.32 (0.39)	<0.0001
Impaired glucose tolerance (yes/no)	53 (20.0)	22 (1.8)	<0.0001	34 (12.45)	14 (1.22)	<0.0001
Uric acid (μmol/L)†	308.75 (87)	255 (73.5)	<0.0001	400.5 (106)	339 (87.5)	<0.0001
Total cholesterol (mmol/L)	7.22 (1.19)	6.57 (1.3)	<0.0001	6.75 (1.17)	6.46 (1.1)	0.0003
LDL-cholesterol (mmol/L)	4.84 (1.09)	4.29 (1.18)	<0.0001	4.50 (1.05)	4.36 (0.99)	0.04
Daily smoking (yes/no)	59 (21.6)	364 (28.6)	0.03	73 (26.3)	333 (29.0)	0.4
Measurements of atherosclerosis						
Baseline mean IMT(mm)	0.85 (0.16)	0.77 (0.15)	<0.0001	0.87 (0.17)	0.83 (0.17)	0.0008
Plaque presence (%)	132 (48.4)	383 (31.0)	<0.0001	137 (49.3)	484 (421)	0.03
Baseline TPA (mm^2^)†	7.84 (13.75)	4.55 (9.66)	<0.0001	9.48 (13.55)	7.75 (13.58)	0.05
Use of medication						
Antihypertensive (yes/no)	59 (21.6)	80 (6.5)	<0.0001	44 (15.9)	73 (6.4)	<0.0001
Lipid-lowering (yes/no)	20 (7.3)	3 (0.2)	<0.0001	28 (10.7)	6 (0.59)	<0.0001
Antidiabetic (yes/no)	10 (3.7)	4 (0.39)	<0.0001	6 (2.2)	4 (0.4)	0.001

* Numbers are means (SD) or numbers (%), † median (interquartile range).

HDL; high-density lipoprotein cholesterol, LDL; low-density lipoprotein cholesterol. HbA1c; glycosylated hemoglobin, IMT; intima-media thickness, TPA; total plaque area.

The Tromsø Study.

**Figure 1:**
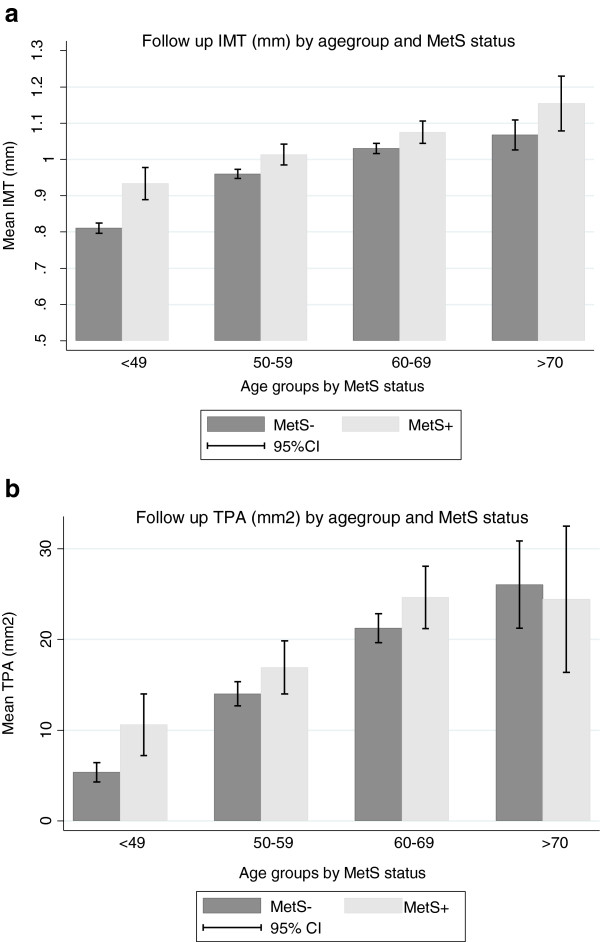
**a: Mean IMT (mm) at follow up in subjects with and without metabolic syndrome (MetS), by age group.** Error bars represent 95% confidence intervals. b: Mean TPA (mm²) at follow up in subjects with and without metabolic syndrome (MetS), by age group. Error bars represent 95% confidence intervals.

**Figure 2:**
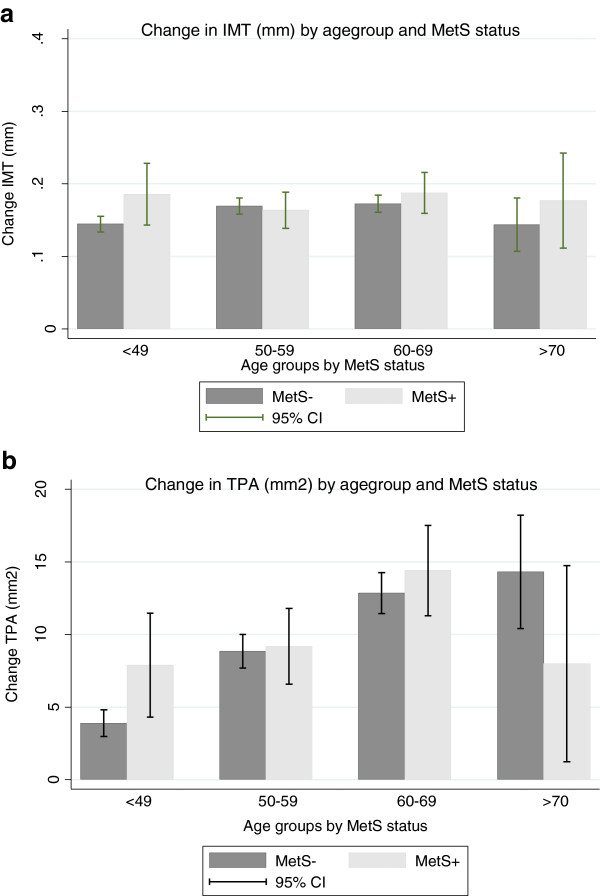
**a: Change in IMT in subjects with and without metabolic syndrome (MetS), by age group.** Error bars represent 95% confidence intervals. b: Change in TPA in subjects with and without metabolic syndrome (MetS), by age group. Error bars represent 95% confidence intervals.

Mean observation time was 13.2 years. Follow-up levels of IMT and TPA were higher in subjects with MetS than in controls, most pronounced in those below 70 years of age. Change in IMT and TPA was associated with Mets only in those younger than 50 years (Table 2, Figures 1 and 2).

**Table 2:** Carotid atherosclerosis after 13-years in subjects with and without metabolic syndrome, by age

	**Metabolic syndrome**					**Metabolic syndrome**				
	**Yes**		**No**			**Yes**		**No**		
Age, years	N	IMT, mm	N	IMT, mm	P*	N	TPA, mm^2^	N	TPA, mm^2^	P*
0-49	81	0.940	483	0.818	<0.0001	79	10.748	479	5.737	0.0001
50-59	224	1.014	965	0.964	<0.0001	218	18.146	956	14.569	0.02
60-69	213	1.088	835	1.041	0.008	209	25.494	830	21.814	0.06
≥70	32	1.135	101	1.059	0.09	31	28.647	99	24.543	0.46
Total	550	1.039	2384	0.966	<0.0001	537	20.524	2364	15.740	<0.0001
Age, years	N	ΔIMT, mm	N	ΔIMT, mm	P*	N	ΔTPA, mm^2^	N	ΔTPA, mm^2^	P*
0-49	81	0.185	483	0.143	0.009	79	7.858	477	4.031	0.0006
50-59	222	0.169	965	0.160	0.5	218	9.857	953	9.203	0.6
60-69	208	0.195	832	0.176	0.2	208	15.363	826	13.116	0.4
≥70	32	0.157	101	0.126	0.5	31	10.951	99	11.227	0.9
Total	543	0.178	2381	0.165	0.13	536	11.763	2355	9.613	0.02

* p for differences between subjects with and without metabolic syndrome.

IMT; intima-media thickness at follow-up, TPA; total plaque area at follow-up, ΔIMT; change in intima-media thickness from baseline to follow-up, ΔTPA; change in total plaque area from baseline to follow-up.

The Tromsø Study.

In stepwise multiple regression analysis, MetS was independently associated with follow-up IMT (β = 0.051 mm, p < 0.0001) and TPA (β = 0.372 mm^2^, p = 0.009) in men. MetS predicted follow-up IMT (β = 0.045 mm, p = 0.001) in women only (Table 3). In analyses stratified by age, MetS predicted progression of IMT (β = 0.043 mm, p = 0.046) and TPA (β = 1.02 mm^2,^ p = 0.002) in men below 50 years of age, but not in the total population.

**Table 3:** Associations* between metabolic syndrome and carotid atherosclerosis after 13 years

	**Follow-up levels**				**Change from baseline to follow-up**			
	**IMT (mm)**		**TPA**^**†**^**(mm²)**		**ΔIMT (mm)**		**ΔTPA**^**†**^**(mm²)**	
	**β**	**p**^**‡**^	**β**	**p**^**‡**^	**β**	**p**^**‡**^	**β**	**p**^**‡**^
**Men**								
Metabolic syndrome	0.051	0.0003	0.372	0.009	-	-	-	-
Age	0.008	<0.0001	0.074	<0.001	-	-	0.031	<0.0001
LDL cholesterol	0.016	0.004	0.232	<0.001	-	-	-	-
Daily smoking	0.038	0.002	0.564	<0.001	-	-	0.514	0.001
**Women**								
Metabolic syndrome	0.045	0.0004	**-**	**-**	-	-	-	-
Age	0.008	<0.0001	0.049	<0.0001	0.002	<0.0001	0.031	<0.0001
LDL cholesterol	0.022	<0.0001	0.227	<0.0001	-	-	0.204	0.0002
Daily smoking	-	-	0.503	<0.0001	0.033	0.001	0.498	0.0002

*Stepwise multivariable linear regression analysis with forward selection and significance level 0.05 for entry into the model.

†square-root-transformed values were used in the analyses.

‡p values for β-coefficients.

IMT; intima-media thickness, TPA; total plaque area, LDL; low-density lipoprotein cholesterol.

The Tromsø Study.

Uric acid level (log-transformed) was not independently associated with IMT or TPA in multivariable analyses, and further adjustment for uric acid did not change did not change the estimates. Adjustment for lipid-lowering, antiplatelet and antihypertensive treatment at follow-up weakened the relationship between MetS and follow-up levels of IMT and TPA, but not substantially.

In stepwise multivariable analyses with each component of the MetS entered separately and adjusted for age, LDL-cholesterol and smoking, hypertension was consistently associated with follow-up levels of TPA and IMT in both sexes and with progression of TPA in women (Table 4). Low HDL-cholesterol levels were associated with follow-up levels of IMT women. Impaired glucose tolerance was associated with follow-up levels of IMT and TPA and with progression of IMT in men. Hypertriglyceridemia was associated with follow up levels of IMT in both men and women, but not with progression. We found no association between abdominal obesity and IMT or TPA.

**Table 4:** Associations* between components of metabolic syndrome and carotid atherosclerosis after 13 years

	**Follow-up levels**				**Change from baseline to follow-up**			
	**IMT (mm)**		**TPA**^**†**^**(mm²)**		**ΔIMT (mm)**		**ΔTPA**^**†**^**(mm²)**	
	**β**	**p**^**‡**^	**β**	**p**^**‡**^	**β**	**p**^**‡**^	**β**	**p**^**‡**^
**Men**								
Age	0.008	<0.0001	0.102	<0.0001	-	-	0.031	<0.0001
Components of MetS								
Hypertension	0.045	0.0004	0.642	0.0003	-	-	-	-
Abdominal obesity	-	-	-	-	-	-	-	-
Hypertriglyceridemia	0.029	0.01	**-**	**-**	-	-	-	-
Low HDL-level	-	-	-	-	-	-	-	-
Impaired glucose tolerance	0.102	0.001	1.129	0.01	0.075	0.006	-	-
LDL cholesterol	0.013	0.02	0.263	0.0006	-	-	-	-
Daily smoking	0.04	0.0001	1.134	<0.0001	0.021	0.006	0.516	0.001
**Women**								
Age	0.008	<0.0001	0.073	<0.0001	0.002	<0.0001	0.027	<0.0001
Components of MetS								
Hypertension	0.041	<0.0001	0.643	<0.0001	-	-	0.308	0.02
Abdominal obesity	-	-	-	-	-	-	-	-
Hypertriglyceridemia	0.026	0.014	-	-	-	-	-	-
Low HDL-level	0.031	0.012	-	-	-	-	-	-
Impaired glucose tolerance	-	-	-	-	-	-	-	-
LDL cholesterol	0.021	<0.0001	0.425	<0.0001	-	-	0.195	0.0004
Daily smoking	0.025	0.025	0.956	<0.0001	0.034	0.0008	0.537	<0.0001

* Stepwise multivariable linear regression analysis with forward selection and significance level 0.05 for entry into the model.

†square-root-transformed values were used in the analyses.

‡p values for β-coefficient.

MetS; metabolic syndrome, HDL; high-density lipoprotein cholesterol, LDL; low-density lipoprotein cholesterol.

The Tromsø Study.

## Discussion

The main finding of our study was that MetS was an independent predictor of follow-up IMT and TPA in men and women. MetS was an independent predictor of progression of IMT and TPA in subjects below 50 years of age, but not in other age groups.

Our finding of increased IMT in subjects with MetS after 13 years of follow-up is in line with results from previous cross-sectional studies [1,3,8,9]. Longitudinal data are scarce. In a posthoc analysis on 2334 hypertensive patients in the European Lacidipine Study on Atherosclerosis (ELSA), progression of IMT was slightly greater in patients with MetS, but this was not significant after adjustment for other cardiovascular risk factors [20]. In our study, change in IMT and TPA was most pronounced in younger age groups. This is in line with the results from a population-based study of 1809 young Finns aged 32 ± 5 years, where MetS was associated with progression of IMT in subjects aged 24–39 years [10]. We found no association in the older age groups. This may indicate that MetS is more important for the early stages of the atherosclerotic process, a process which accelerates in the 4^th^ to 5^th^ decade. However, in a study on 102 elderly women, incident MetS predicted progression of IMT after 12-years follow-up [21].

Few studies have assessed the relationship between MetS and plaque measurements [6,9,11,22]. In a multi-ethnic cross-sectional study, MetS and the number of MetS components was independently associated with plaque presence [22]. A prospective study on 166 Cree-Indians showed that MetS at baseline predicted follow-up levels of IMT, but not total plaque volume, a measure which is strongly correlated with TPA. However, change in IMT and total plaque volume was not assessed. In the Bruneck study, MetS was associated with 5-year change in atherosclerosis as assessed by novel plaque and stenosis formation [11].

Previous studies found no clear evidence that MetS predicted IMT progression better than expected from the sum of the individual components [10]. In our study, hypertension was the one component most consistently associated with follow-up levels of carotid atherosclerosis among men and women. Hypertension was also independently associated with progression of TPA in women. Impaired glucose tolerance was associated with follow up IMT and progression of IMT in men. In a systematic review, three of nine of cross-sectional studies found significantly larger IMT in subjects with impaired glucose tolerance [23]. Both low HDL-levels and hypertriglyceridemia were associated with follow-up levels of IMT and TPA, but not with progression of atherosclerosis.

Increased use of medication that may influence the atherosclerotic process during follow-up could have confounded our results. Use of lipid-lowering, antiplatelet and antihypertensive drugs increased during follow-up, most pronounced for use of lipid-lowering drugs (from 1.9% to 26.9%). The association between MetS and IMT and TPA was somewhat weakened with adjustment for use of medication at follow-up, but not substantially, and this could not explain the lack of association between MetS and progression of atherosclerosis.

In a previous study, serum uric acid level was associated with MetS and carotid atherosclerosis in patients diagnosed with diabetes mellitus type 2 [24]. We found no independent association between serum uric acid and carotid atherosclerosis in our population-based study. Possible links between metabolic dysfunction and atherosclerosis may be secretion of adipokines by adipose tissue. Several adipokines have been reported to promote arterial stiffness, inflammation and atherosclerosis in subjects with diabetes and coronary heart disease [25-27]. Adipokines were not measured in the Tromsø Study.

In general, it is more difficult to detect associations between risk factors and change in atherosclerosis as opposed to single measurements [15,28]. Measurements of progression of atherosclerosis are more prone to errors than single measurements because random measurement errors at baseline and follow-up are accumulated. This can attenuate the differences aimed to be detected, and may preclude the detection of a positive relationships between MetS and *change* in atherosclerosis as opposed to single measurement of atherosclerosis at follow-up.

Our study has some important limitations. As observed in many other large population-based epidemiological studies, the overall attendance rates of the Tromsø Study fell from 77% in 1994–1995 to 64% in 2007–8 [12]. The attendance at follow-up was lower in those with MetS at baseline. During follow-up, the proportion that moved from Tromsø was lower in the MetS group compared to the non-MetS group (5.9% vs. 7.9%, p = 000.4), but this was by far outweighed by selection bias due to higher mortality in those with than without MetS (28.8% vs 19.6%, p <0.0001). Further selection bias may have occurred due to higher morbidity in the MetS group [12]. Furthermore, the attendance rates at follow-up were low in subjects ≥70 years, which calls for caution in making inferences about this group.

## Conclusion

In conclusion, we found that MetS was associated with IMT and TPA levels at follow up. In analyses of the different components of MetS, hypertension showed the most consistent positive association with carotid atherosclerosis. MetS was associated with progression of IMT and TPA only in those below 50 years of age. The results may indicate that MetS may be involved in the initiation of the atherosclerotic process.

## Abbreviations

MetS, Metabolic syndrome; NCEP, ATPIII (National Cholesterol Education Programme, Adult Treatment Panel III); TPA, Total plaque area; IMT, Intima-media thickness; HDL, Serum high density lipoprotein; HbA1C, Glycosylated haemoglobin; CCA, Common carotid artery; ICA, Internal carotid artery; CVD, Cardiovascular disease; CCA-FW-IMT, Common carotid far wall intima-media thickness; CCA-NW-IMT, Common carotid near wall intima-media thickness; AMS, artery measurement system; CV, Coefficient of variation.

## Competing interests

We declare that we have no competing interests.

## Authors’ contributions

MH acquired the carotid ultrasound data, performed the statistical analysis, and drafted the manuscript. KAA acquired the carotid ultrasound data, and made critical revision of the manuscript. SHJ participated in the design of the study, and made critical revision of the manuscript. EBM designed and coordinated the study, acquired the carotid ultrasound data, handled funding, and helped to draft the manuscript. All authors read and approved the final manuscript.
