# Correction: The metabolic syndrome and progression of carotid atherosclerosis over 13 years: the Tromsø study

**DOI:** 10.1186/1475-2840-12-144

**Published:** 2013-10-31

**Authors:** Marit Herder, Kjell Arne Arntzen, Stein Harald Johnsen, Ellisiv B Mathiesen

**Affiliations:** 1 Department of Community Medicine, University of Tromsø, Tromsø N-9038, Norway; 2 Department of Radiology, University Hospital North Norway, Tromsø, Norway; 3 Department of Neurology and Neurophysiology, University Hospital North Norway, Tromsø, Norway; 4 Department of Clinical Medicine, University of Tromsø, Tromsø, Norway

## 

After publication of our work, we have noticed some inadvertent errors in the article [1]. We deeply regret that these occurred and are hereby presenting corrections.

In the 'Subjects’ section (page 2), the second paragraph should read as follows from the fifth sentence and onwards:

“During follow-up, 1515 persons died and 468 moved from Tromsø. Of the remaining 4744 subjects who were still alive and living in Tromsø, 2974 subjects (62.6%) attended the carotid ultrasound examination in the 6th survey in 2007–2008, and were included in the present study.”

In the 'Statistical analysis’ section (page 4), the sixth and seventh sentences should read: “Linear regression models were fitted with IMT and TPA as dependent variables and MetS, age, LDL cholesterol and smoking as independent variables. Similarly, stepwise linear multivariable models with forward selection and significance level 0.05 for entry into the model were fitted with each component of the metabolic syndrome entered as separate independent variables, together with age, LDL cholesterol and smoking.”

In Table 2, the correct value for the ΔIMT value in participants with metabolic syndrome in the age group 50–59 years was 0.160 mm. The corresponding value for participants in the same age group without metabolic syndrome was 0.169 mm.

Errors had also occurred during preparation of Figures 1 and 2, and corrected figures are presented here.

The errors had no effect on the scientific content and conclusions.

**Figure 1:**
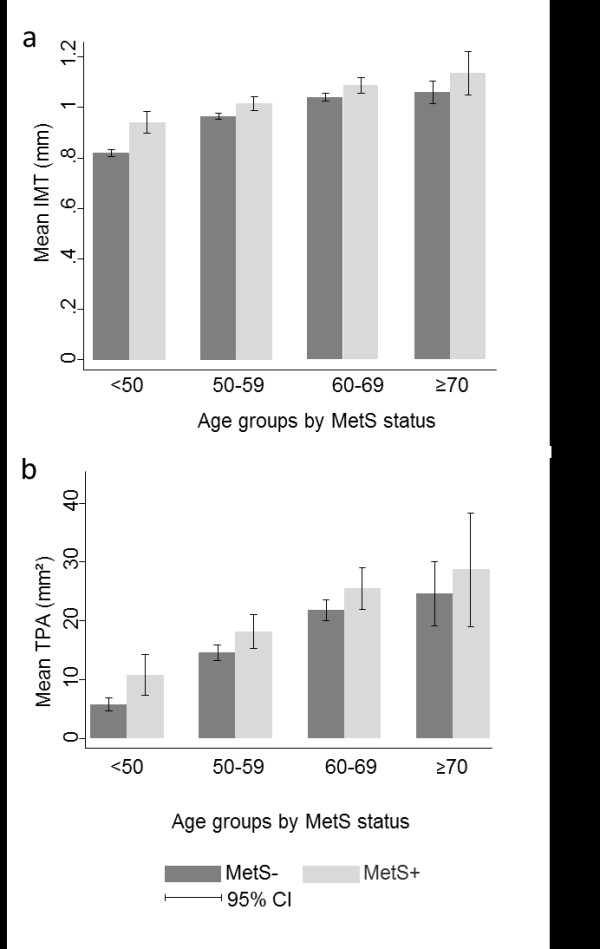
**Follow-up levels of mean intima-media thickness (IMT) and total plaque area (TPA). The Tromsø Study. a**: Mean IMT (mm) at follow-up in subjects with and without metabolic syndrome (MetS), by age group. **b**: Mean TPA (mm^2^) at follow-up in subjects with and without metabolic syndrome (MetS), by age group. Error bars represent 95% confidence intervals (CI).

**Figure 2:**
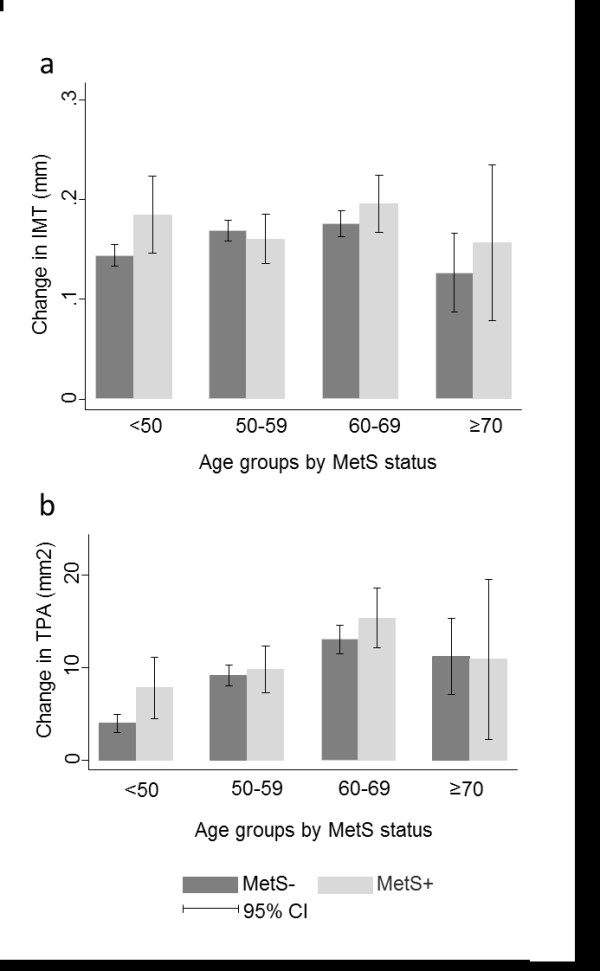
**Change in intima-media thickness (IMT) and total plaque area (TPA) from baseline to follow-up. The Tromsø Study. a**: Change in IMT (mm) in subjects with and without metabolic syndrome (MetS), by age group. **b**: Change in TPA (mm^2^) in subjects with and without metabolic syndrome (MetS), by age group. Error bars represent 95% confidence intervals (CI).
